# How to safely use a portable electric generator

**Published:** 2011-09

**Authors:** Pak Sang Lee, Ismael Cordero

**Affiliations:** Technical Administrator, Division of Epidemiology & Genetics, UCL Institute of Ophthalmology & Moorfields Eye Hospital, 11-43 Bath Street, London EC1V 9EL, UK; Formerly Senior Clinical Engineer, ORBIS International. Email: ismaelcordero@me.com

**Figure:**
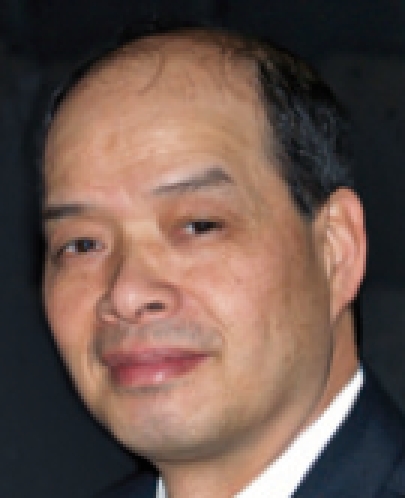


**Figure:**
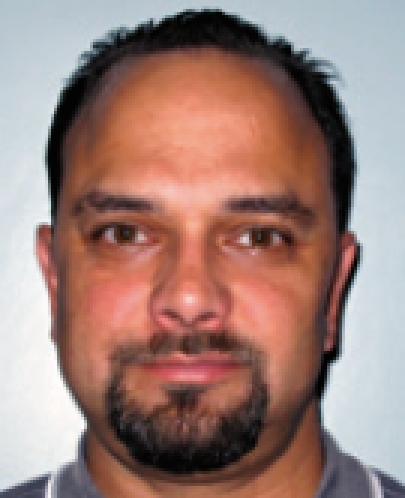


If your facility does not have a permanent stand by generator to use for temporary power outages, or if you are conducting mobile clinic activities in areas without electrical power, you may need a portable generator to power your electrical devices.

A generator that isn't powerful enough may overheat and catch fire; it can also damage equipment. To calculate the generator capacity you need, add the wattage of all the equipment you want to use at the same time (you can find this on the equipment's label or in its manual). The stated ‘running rating’ or ‘continuous rating’ of the generator must be at least 1.3 times higher than this figure. This will ensure that you are running the generator at no more than 75% of its capacity, which is efficient and will prolong the generator's life.

Use a separate voltage regulator and a surge suppressor in order to guarantee stable and ‘clean’ voltage to your delicate eye care equipment. The capacity of the voltage regulator and surge suppressor should be calculated in the same manner as described for the generator.

## Using your generator

You will need:

- Fuel (consult the generator manual)
- Oil (recommended by manufacturer)
- Cable reel
- Voltage regulator
- Surge suppressor socket strip
- Any other socket strips needed to plug in your eye equipment.

## Starting the generator

**Figure 1:**
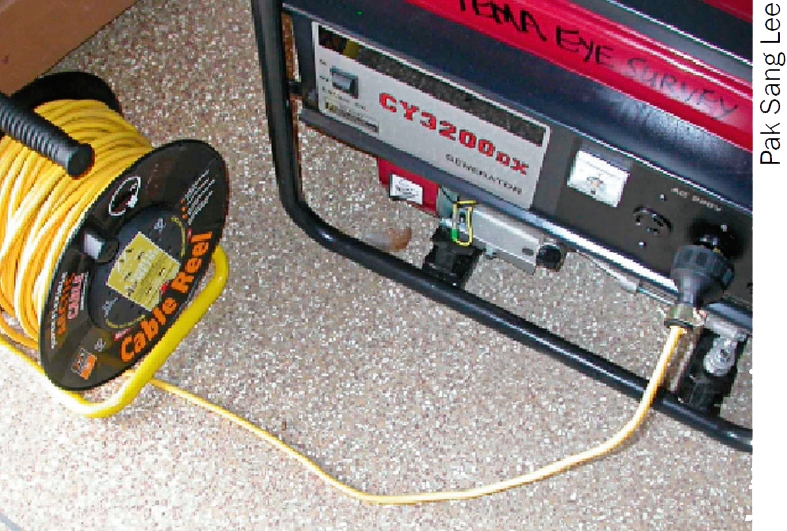
Connecting the cable reel to the generator. Note: unroll the cable reel before you plug the voltage regulator into the cable reel socket

**Figure 2:**
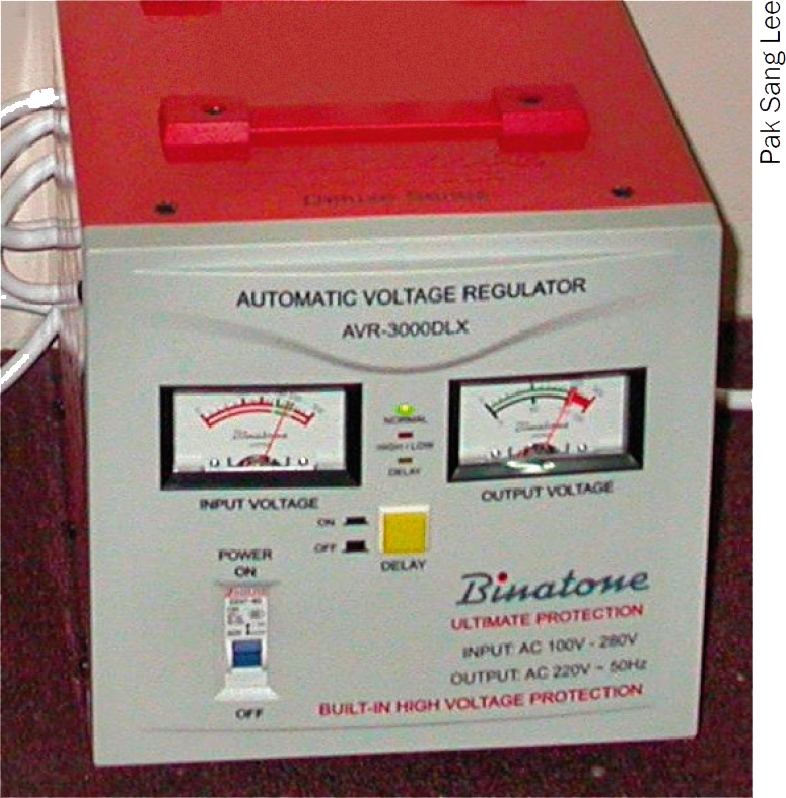
Voltage regulator

**Figure 3:**
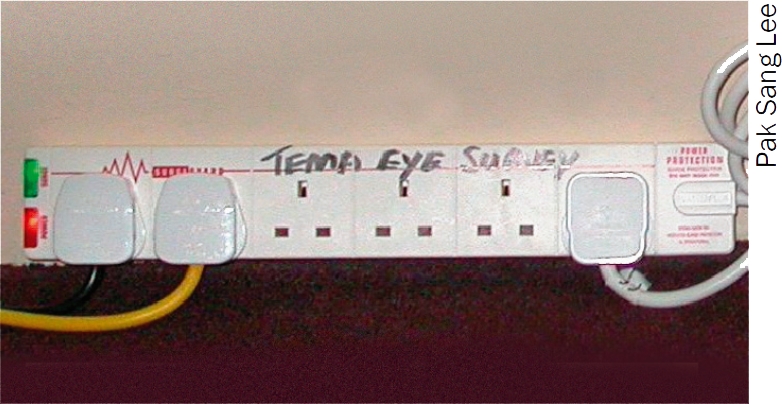
Surge supressor socket strip

1. Fill the fuel tank and check the fuel indicator to ensure it is full. Consult the manual to find out how long the generator will run on a full tank.
2. Check the oil level. Use only the type of oil recommended by the manufacturer.
3. Switch on the fuel.
4. Switch on the choke. Only use the choke when the engine is cold.
5. Make sure the voltage regulator is disconnected from the cable reel. Connect the plug from the cable reel to the generator socket (Figure 1).
6. Switch the generator's rocker switch to the ‘on’ position. Pull the starting cord swiftly towards you. You may have to do this a few times before the engine starts.
7. Once the engine starts and has stabilised, slowly move the choke lever back to the ‘off’ position.
8. Connect the voltage regulator (Figure 2) to the cable reel socket and turn it on. Make sure the cable is unrolled, that is, all the cable is out of the cable reel. This will prevent overheating.
9. Connect the surge suppressor socket strip (Figure 3) to the voltage regulator and turn it on.
10. Plug in other socket strips. Now plug in your ophthalmic equipment.

**Figure 4:**
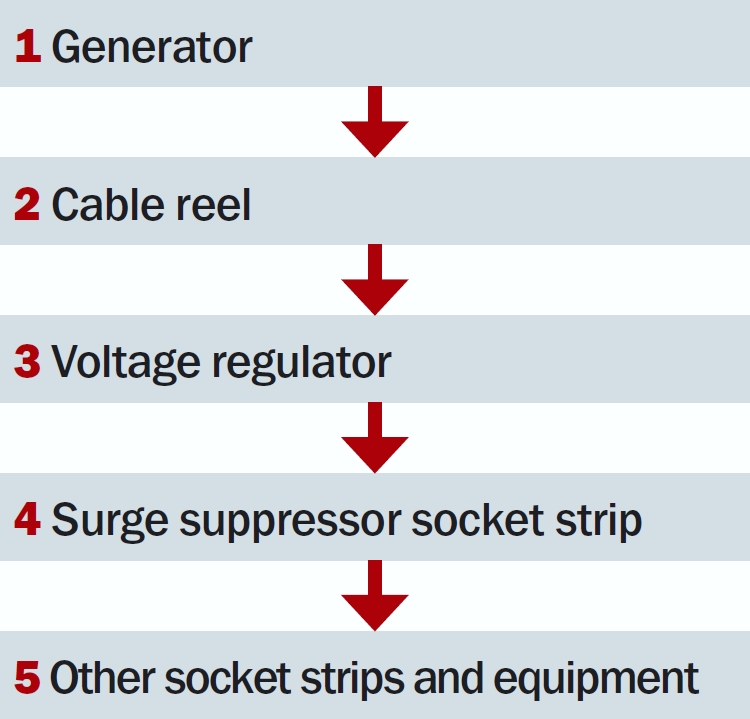
Connection sequence. Connect devices in the order shown above. Before powering on any device, make sure that the device preceding it in the sequence is connected and turned on

## Stopping the generator

1. Switch off all the equipment.
2. Switch off the surge suppressor power socket strips.
3. Switch off the voltage regulator.
4. Switch off the generator by pushing the generator's rocker switch to the ‘off’ position.

## General operation and safety tips

- Keep your generator in a level, clean, and dry environment.
- Never refuel your generator while it is running since fuel spilled into a running engine can cause fires.
- Never operate a portable generator inside a building or near windows and doors. It produces deadly carbon monoxide gas.
- Most generators are very noisy. Make sure that the generator is placed as far away from the eye care unit as possible so that it does not disrupt patient care.
- Keep your generator ready for use by testing it on a regular basis (once a month is a good rule of thumb).
- On electric start models, charge the battery on a regular basis.
- Keep fresh fuel on hand at all times, and follow safety advice on storing and using fuels. Gasoline and biodiesel have a shelf life of about six months; diesel lasts for about a year. * Engine parts get very hot during operation. Severe burns may result if touched.
- Never attempt to repair an electric generator. Only a qualified service technician should perform repairs.

**Note**: improper use or installation of an electric generator can cause property and equipment damage, serious injury, and even death.

